# Battle of the Sexes in Best of Breed: Sex Influences Dogs’ Success in the Show Ring

**DOI:** 10.3390/ani8120240

**Published:** 2018-12-18

**Authors:** Bethany J. Wilson, Alicia J. Kasbarian, Navneet Dhand, Paul D. McGreevy

**Affiliations:** Sydney School of Veterinary Science, Faculty of Science, The University of Sydney, Sydney, NSW 2006, Australia; bethany.wilson@sydney.edu.au (B.J.W.); akas5676@uni.sydney.edu.au (A.J.K.); navneet.dhand@sydney.edu.au (N.D.)

**Keywords:** purebred dogs, breeding, dog show, conformation

## Abstract

**Simple Summary:**

When breeding dogs, the dam and the sire contribute equally to the genetics of each of their puppies, and so each parent should be equally important to show-ring success. The current study collated samples of dog show results to explore relationships between sex and the likelihood of success in the show ring. It focused on toy and giant breeds to explore any differences in equity, if it existed, at either end of the size spectrum. It revealed that the rate at which male and female dogs were exhibited were similar but that male dogs were a significant advantage of winning Best of Breed titles.

**Abstract:**

Much of the research on pedigree dog breeding has been directed towards understanding the implications of reduced genetic diversity and the prevalence of inherited disorders. An example is the potential role of the popular sire effect in perpetuating genetic defects. If male dogs are more likely than bitches to be identified as examples of members of a breed that align with breed standard, they may be selected for breeding earlier. This may contribute to the influence of individual males and contribute to popular sire effect. Conversely, if breed standards are written in a sex-neutral fashion, and if dogs are entered, exhibited, and judged in a sex-neutral fashion, then we would expect the success of female dogs in the show ring to be equal to that of their male counterparts. With a focus on toy and giant breeds, the current pilot study collated samples of dog show results to explore relationships between sex and the likelihood of success in the show ring. It focused on toy and giant breeds to explore any differences in equity, if it existed, at either end of the size and concomitant age-at-maturation spectrum. For the purpose of this study, toy breeds were those that weigh < 10 kg at maturity while giant breed dogs were those that exceed 45 kg. Within these two clusters, the least (n = 3) and most popular (n = 3) breeds were then selected to explore any potential role of sex on success in the show ring. The popularity of breeds was determined using the numbers of dogs registered with the Australian National Kennel Council. Using results from dog shows (n = 18) from 2015 to 2016, data on 1,080 dogs were obtained. Within these 12 breeds for the 18 shows, there were 137 Best of Breed (BOB) titles awarded: Pug (n = 18), Toy Poodle (n = 18), Bullmastiff (n = 14), Rottweiler (n = 17), Fox Terrier (Smooth) (n = 18), Bloodhound (n = 3), Schnauzer (miniature) (n = 15), Great Dane (n = 17), Norfolk Terrier (n = 10), Norwich Terrier (n = 5), Central Asian Shepherd Dog (n = 2). Despite the near parity of male and female dogs being exhibited, of these 137 titles, 86 (62.8%) were awarded to male dogs (at least 41 individuals) and 51 (37.2%) to female dogs (at least 32 individuals) showing that male dogs are more likely to win BOB titles (χ^2^ = 9.4455, df = 1, *p*-value = 0.002117). Among the toy subset of breeds, this effect was higher (χ^2^ = 6.798, df = 1, *p*-value = 0.009126) than among the giant breed subset, for whom the advantage to male dogs did not reach statistical significance versus χ^2^ = 3.0967, df = 1, *p*-value = 0.07845). This suggests that judges find the male dogs more appealing, presumably because they are more aligned with breed standards.

## 1. Introduction

Many studies have shown the deleterious effects of unchecked purebred dog breeding practices [1,2,3]. Arguably, while formal dog breeding has produced an impressive diversity of breed shapes and sizes [4], it has also resulted in reduced genetic variation within breeds [5,6]. Much of current literature discusses the issues of breeding to conform to breed standards (or aesthetics) [7,8], specifically the phenotypic and genotypic consequences of selective breeding [8,9], as well as the prevalence of inherited disorders [2]. While it is acknowledged that not all inherited disorders are based on conformation and aesthetics, Summers et al. [10] identified over 312 nonconformation-linked disorders of pedigree dogs that seem to be consequences of breeding within a closed studbook and selective breeding pressures.

The dangers of breeding too many litters from young dogs—the so-called popular sire effect—is another common issue in dog breeding [5]. This phenomenon is characterised by the relative overuse of popular champion dogs to the exclusion of other males [10]. In their study, Leroy & Baumung [11] demonstrated that, depending on the breed in question, as few as 33% of the total number of registered sires standing at stud are used by breeders. This is concerning when one considers that, in contrast to senior studs, more extensively used popular young sires carry the risk of disseminating as yet unknown inherited disorders or genetic defects [2,11]. It is worth emphasising that ethical breeders are unlikely to adopt such questionable breeding practices but the temptation to make the most of a dog that is well regarded in the show ring is undeniable. 

An Australian case study of 32 dog breeds demonstrated diversity within some breeds, attributed to conscientious breeders making informed decisions that helped to avoid the popular sire effect [12]. However, to focus on the sires alone, as much of the current literature does, would be to overlook the maternal contribution to future generations. Plainly, significant genetic gains can be achieved through careful selection of not only stud dog but also the dam. Some studies (e.g. Czerwinski et al. 2016 [13]), suggest that information on dam selection for breeding is poor, and that dams are often selected primarily on their temperament and level of maternal care.

Under the rules imposed by breed authorities in a laudable bid to assure the welfare of breeding stock, registered purebred dog breeders are often restricted in the use of their bitches in breeding programs. From an animal welfare standpoint, these restrictions provide prudent limitations on the number of litters a bitch can carry and some associated age restrictions (time of first breeding, maximum number of litters, frequency of litters, and time interval between whelpings). However, if one has a focus on genetic gain, these restrictions limit the capacity of bitches to make genetic contributions when compared with stud dogs. Thus, bitches have reduced generational turnover compared to their male counterparts [14]. 

Notwithstanding the reduced capacity of bitches to reproduce and contribute genetics towards future generations, if differences in size, and therefore the latency to maturation [15], are taken into consideration, the generation interval is also shorter in larger breeds of dogs (especially giant breeds) when compared with toy breeds. Assessment of body size relative to lifespan demonstrated that larger breeds of dogs have a reduced lifespan due to the accelerated rate at which they age after reaching maturity [16]. 

Strictly speaking, success in the show ring should be when dogs align best with breed standards [17] and, theoretically at least, this point in time should not be influenced by factors such as age, breed popularity, or sex [18].

Although the dam and the sire contribute equally to the genetics of each of their puppies, dog breeders may use very different criteria for the selection of male and female parents. For example, a recent study by Czerwinski et al. [13] investigated the attitudes and priorities of registered dog breeders towards dam and sire selection. The participants in this study indicated that, in sire selection, conformation, size, pedigree, and temperament are the primary considerations. In contrast, information on dam selection for breeding is unclear but suggests that dams are often selected primarily on their temperament and level of maternal care [13].

While temperament and maternal care are critical to both the short- and long-term well-being of their offspring, selection of female parents only on these traits limits the abilities of breeders to make full use of both the genetic merit and genetic diversity that reside equally among the male and female members of the breed.

Despite living in an era when breeders have unparalleled access to educational resources, and the ability to share information, it seems that many are not yet able to fully leverage the genetic merit and diversity of their female breeding dogs. We hypothesized that one contributing factor may be an impression, among some breeders, that the genetic merit of the breed is found more reliably in male dogs than in female dogs, and that a differential in success in the show ring may be partly responsible for this.

If the breed standards are written in a sex-neutral fashion, and if dogs are entered, exhibited, and judged equitably, then we would expect that the success of female dogs in the show ring would be equal to that of their male counterparts. In contrast, if male dogs have significantly greater success in the show ring, this would suggest a sex bias at one or more levels of the exhibition process. Such an outcome may, in turn, create false impressions that the male phenotype is the “true” breed phenotype and thus risk the underutilization of that genetic merit and diversity that resides in the breed’s female members.

The current pilot study explored samples of dog show results to explore relationships between sex and the likelihood of success in the show ring. It focused on toy and giant breeds to explore any differences in equity, if it existed, at either end of the size spectrum. We selected toy and giant breeds because we were interested in how the difference with which they reach skeletal maturity may influence the number of male and female winners for each breed. 

## 2. Materials and Methods 

Results of New South Wales (NSW) dog shows (n = 18) from 2015 to 2016 were obtained through completed/recorded dog show schedules uploaded and made available through social media. The reliance on extant data meant that ethics committee approval was not required. The data were pooled from shows held within the metropolitan Sydney area over this period. To reduce any potential confounding factor of local bias, show results were from only those club shows that consisted of a full international judging panel. Data extracted from the dog show catalogues included information on the sex, date of birth (to determine age), and breed of all exhibits entered and the winner of the Best of Breed (BOB) award, as well as any of these dogs that placed first or second in their Best in Group (BIG) award.

Data on exhibits (dogs) were included only for those individuals eligible for championship points. This means that the individual had to be competing in the minor class and be a minimum of 6 months of age. Data were excluded if exhibits had been absent on the day of the show, and data were reported as missing in cases where exhibits were non-awarded by the judge. Non-awarded dogs are those considered by the judged to be incorrect to type, based on the breed standard or otherwise possessing a dismissible breed fault.

A selection of “toy” (<10 kg mature weight) and “giant” (>45 kg) breeds of dogs was used for this project based on the method used by Teng et al. [4]. The least (n = 3) and most popular (n = 3) breeds that represent these types of dogs were selected. The three most popular and three least popular breeds of dog were identified on the basis of their cumulative number of registrations across the 2013–2016 period (inclusive) using the published Australian National Kennel Council National Animal Registration Analysis data. The breeds selected by this process are shown in Table 1.

R’s prop.test function was used to test the hypothesis that one proportion (e.g., proportion of BOB entrants who are male) was equal to the proportion of males in another group (e.g., proportion of BOB winners who are male) or with the presumed proportion of males in the total population (i.e., 0.5). The function calculates χ^2^ estimates with Yates’ continuity correction and associated *p* values. For this study, alpha was taken as 0.05.

## 3. Results

The over-representation of male dogs among Best of Breed (BOB) and Best in Group (BIG) records is shown in Table 2.

Among all dog exhibits, 523 out of 1080 (48.4%) were males, representation which did not differ significantly from the presumed population 50% (χ^2^ = 1.0083, df = 1, *p*-value = 0.3153). Among the subgroups selected, this held true for toy breeds (49.9%, χ^2^ = 0.0012438, df = 1, *p*-value = 0.9719), and for giant breeds (44.2%, χ^2^ = 3.4819, df = 1, *p*-value = 0.06), as well as for the less popular breeds (54.3%, χ^2^ = 1.2228, df = 1, *p*-value = 0.2688) and for the more popular breeds (47.2%, χ^2^ = 2.6797, df = 1, *p*-value = 0.1016).

### 3.1. Best in Breed

Despite the near parity of male and female dogs being exhibited, male dogs were significantly overrepresented among Best of Breed winners. Within these 12 breeds for the 18 shows, there were 137 Best of Breed Titles awarded, as follows: Pug (n = 18), Toy Poodle (n = 18), Bullmastiff (n = 14), Rottweiler (n = 17), Fox Terrier (Smooth) (n = 18), Bloodhound (n = 3), Schnauzer (miniature) (n = 15), Great Dane (n = 17), Norfolk Terrier (n = 10), Norwich Terrier (n = 5), Central Asian Shepherd Dog (n = 2). The most BOB titles among the 12 breeds at a single show was 9 (7 of the shows) and the fewest was 5 (1 show).

Of these 137 titles, 86 (62.8%) were awarded to male dogs (at least 41 individuals), and 51 (37.2%) to female dogs (at least 32 individuals). This is significantly different from the proportion of male and female exhibits (χ^2^ = 9.4455, df = 1, *p*-value = 0.002117) showing that male dogs are more likely to win BOB titles.

Among the 84 BOB titles won by the toy subset, 55 (65.5%) were won by male toy dogs. These had a significantly higher chance of winning a BOB award than a female toy dog (χ^2^ = 6.798, df = 1, *p*-value = 0.009126). Among the giant breed subset, where 31 of the 53 BOB titles (58.5%) were won by males, the advantage associated with being male did not reach statistical significance (χ^2^ = 3.0967, df = 1, *p*-value = 0.07845).

Among the “less popular” subset of breeds, 27 of their 38 BOB titles (71.1%) went to male dogs, but the advantage to male dogs did not reach statistical significance in this case either (χ^2^ = 2.9402, df = 1, *p*-value = 0.0864.) However, among the “more popular” subset the 59 out of 99 BOB titles (59.6%) awarded to male dogs did represent a statistically significant advantage (χ^2^ = 4.9914, df = 1, *p*-value = 0.02547).

### 3.2. Best in Group

Within these 12 breeds for the 18 shows, there were 19 “Best in Group” (n = 9) or “Runner-up Best in Group” (n = 10). Titles were awarded to only 4 of the 11 breeds, these were: Pug (n = 8), Rottweiler (n = 1), Fox Terrier (Smooth) (n = 7), Great Dane (n = 3).

Of these 19 titles, 15 (78.9%) were awarded to male dogs (at least 11 individuals), and 4 (21.1%) to female dogs (at least 3 individuals). This is statistically different from 50% (χ^2^ = 5.2632, df = 1, *p*-value = 0.02178). The most BIG titles among the 12 breeds at a single show was 3 (at 1 of the shows) and the fewest was 0 (5 of the shows).

Table 2 shows an apparent disproportion in the representation of males between the BOB level and the BIG winner or runner-up level. The increased proportion of males among BIG winners and runners up (78.9%) compared with BIG candidates (i.e. BOB winners, (62.8%)) might suggest that there is still an advantage to male dogs at the level of BIG judging, even after correcting for the advantage at BOB level. However, this did not reach significance (χ^2^ = 1.2693, df = 1, *p*-value = 0.2599).

## 4. Discussion

In Australia, the breed standards are maintained by the Australian National Kennel Council (ANKC). The ANKC’s accredited judges are trained to evaluate the dogs presented for judging in accordance to the breed standards. These standards are a set of guidelines that outline the physical attributes desired for each breed [19]. Based on the judges’ assessments of the exhibits on the day of competition, the dog which best exemplifies the breed standard is awarded BOB and proceeds to compete in the show at higher levels, with dogs of other breeds which were similarly awarded. In this respect, dog show judges are essentially in a position of power to influence which dogs are highly awarded and, while they are extensively trained on the interpretation of a breed standard and impartiality, judges themselves may be influenced by a number of other factors [1,20]. These factors may include: the ‘showiness’ of the dog, the showmanship skills of the handler on the day and the judges’ own personal preference for a particular morphotype of that breed [20]. In this way, subjectivity in judging can have an indirect influence on the selection of breeding stock by promoting particular dogs to the exclusion of other quality dogs [5].

The current data showed that the rate at which male and female dogs were exhibited were similar to what would be expected under parity. Indeed, the minimum number of individual female dogs exhibited (n = 187) was slightly larger than the minimum number of individual male dogs exhibited. Therefore, we did not find any evidence that dog breeders are failing to exhibit female dogs. However, in contrast, we did find that, despite the parity of exhibition, male dogs were a significant advantage of winning BOB titles across the study cohort and among most of its subsets. Providing evidence for the reasons for the current bias is the next logical step. It is possible that judges of a given gender favour one sex of dog over the other. Equally, it may be that breed standards are being observed without bias but that they were inadvertently written with male dogs as the ideal version.

Given that the genome of male and female contemporaries would be similar, these findings suggest four possibilities. First, that the breed standards, when applied impartially, confer an advantage on the male phenotype. This could be tested by blinding judges to the sex of dogs, e.g., using videos of dogs with pixelated secondary sex organs. Second, that judges’ preferences confer an advantage on the male phenotype. It is possible that simply by being bigger [21], males are more appealing to judges. This could be tested by purposefully misleading judges as to the sex of dogs in the afore-mentioned videos. Third, that the mechanics of the show ring somehow confer an advantage on the male candidate for BOB (e.g., being placed in front of the bitch). This could be tested by reversing any biased conventions about how males and females are presented to judges. Finally, that breeders are not showing those female candidates that best represent the breed standards (e.g., they show those bitches they have chosen to breed from, for reasons separate from or in addition to those bitches’ congruency with the breed standards). This could be explored by asking breeders to review and explain their decisions about which females to exhibit.

Plainly, breeders want to breed from the dogs they own. If they wanted to breed from some other dog, they would attempt to acquire that dog. Although this understandable attitude toward breeding is almost entirely separate from activities in the show ring, it must influence the population genetics of any given breed. It may result in breeders breeding all available candidate females. The impact of such attitudes to breeds’ gene pools merit consideration.

As shown by Pedersen et al. [22], selective breeding of particular individuals and bloodlines for showing and competition has contributed to reduced genetic diversity among many dog breeds. Alternatively, overrepresented individuals or specific bloodlines in pedigrees may be contributing to an individual’s success in the show ring. A recent study by Czerwinski et al. [13] investigated the attitudes and priorities of registered dog breeders towards dam and sire selection. The participants in this study indicated that conformation, size, pedigree, and temperament are the primary factors in sire selection. The current pilot study is unique in that it examines Australian conformation judges’ preferences for dogs over bitches at the breed level, in dogs at either end of the size spectrum. 

The likelihood that a dog’s success in the show ring influences breeders’ sire selection merits further scrutiny. On a more global scale, and with the advent of artificial reproductive technologies (e.g., frozen semen) and the ease of which semen can be imported into Australia, breeders have unprecedented access to a wide variety of bloodlines. In some instances, imported semen has been shown to reduce the inbreeding coefficient especially for breeds with a particularly small pool of breeding individuals (e.g., [12]). While this can be considered to have improved genetic diversity through the introduction of new breeding lines, further investigation of its impact on current breeding practices and how successful overseas stud dogs may be represented in the Australian conformation ring is warranted.

Although the current study was able to reveal male advantage at the BOB level, it was not able to detect male advantage at the BIG level, perhaps due to its modest scale, and an analysis of Best in Show was discounted a priori as the data captured a Best in Show awardee from only one of the 18 dog shows. Testing for male advantage using data from more breeds and from more shows over longer time scales would be highly desirable. It would be helpful to explore how judgment bias varies across breeds. For the current pilot study, we selected breeds according to their popularity in registration numbers but we discovered that groupings may not have served as well as intended. Future studies should select dogs according to their distribution at shows rather than registration numbers and ideally would use all represented breeds rather than just six from each grouping. In addition, future analyses of comparable data should examine not just sex percentages, but also the true numbers of individual dogs. If, for example, a significant difference is not found between the number of individual male dogs awarded BOB and the number of individual female dogs awarded BOB, then this may refute the prospect of a sex bias, and instead reflect the persistence of exhibitors trying to get more titles for their dog. Equally, if this proposed analysis reveals that the same dogs are winning BOB in multiple shows, under multiple judges, it would mean that the judges are agreeing. This approach would provide especially valuable information on how well the judges feel individual dogs meet the breed standard.

Additionally, dog show catalogues present a limited amount of information about the exhibits competing in the show on a given day. The data that we were able to extract from these catalogues included the date of birth of the dog (from which we could calculate the age of the dog), sex, breed, and whether or not the dog had been awarded its championship title at the time of entering the show.

Our findings reveal that sex is strongly associated with a dog successfully competing in conformation shows. They indicate the need for further exploration of this topic and its implications for breeding practices [5]. It may be that favouring males in the show ring effectively dilutes or weakens the popular sire effect by creating a larger pool of potential stud dogs for breeders to choose from than would be the case if there was equitable distribution of BOB titles between the sexes. A pragmatic approach to exploring this topic would incorporate a systematic collection of information about pedigree dogs, specifically as data relating to their success in the show ring and how this influences breeding practices, such as which individual dogs are bred from and with whom, as well as their relationship with competition success. This would build on the current finding and provide new evidence on breeding bias and the selection pressures that apply within breeding programs. Exploring breeding practices in this way would reveal any bias breeders may inadvertently apply when selecting dams or sires and potentially provide breed societies and relevant governing bodies with useful information to better guide their breeding decisions. 

## 5. Conclusions

The current findings show that male show dogs in this pilot study are at a significant advantage in the show ring, and are thus more often chosen to represent the champion and thus archetype for that breed. Whether this tendency to equate male with correct type limits the ability of breeders to fully use the genetic merit and diversity within female breeding candidates in Australian populations may merit further investigation, given the current findings.

## Figures and Tables

**Table 1:** The most popular and least popular 12 toy and giant breeds selected for New South Wales (NSW) show results in the current study.

	Giant		Toy		Sum
Popular	Bullmastiff	30	Schnauzer (miniature)	176	896
	Great Dane	188	Pug	332	
	Rottweiler	52	Poodle (Toy)	118	
Rare	Central Asian Shepherd Dog	3	Fox Terrier (Smooth)	143	184
	Bloodhound	3	Norfolk Terrier	24	
	Komondor	0	Norwich Terrier	11	
Sum		276		804	1080

**Table 2:** The proportional distribution of male and female dogs in Best of Breed (BOB) and Best in Group (BIG) classes in selected NSW dog shows (n = 18) from 2015 to 2016.

	Size Category	Male		Female		TOTAL
Exhibits		n	%	n	%	
Bloodhound	Giant	3	100.0%	0	0.0%	3
Bullmastiff	Giant	21	70.0%	9	30.0%	30
Central Asian shepherd dog	Giant	0	0.0%	3	100.0%	3
Fox terrier (Smooth)	Toy	78	54.5%	65	45.5%	143
Great Dane	Giant	69	36.7%	119	63.3%	188
Norfolk terrier	Toy	10	41.7%	14	58.3%	24
Norwich terrier	Toy	9	81.8%	2	18.2%	11
Poodle (Toy)	Toy	47	39.8%	71	60.2%	118
Pug	Toy	161	48.5%	171	51.5%	332
Rottweiler	Giant	29	55.8%	23	44.2%	52
Schnauzer (miniature)	Toy	96	54.5%	80	45.5%	176
TOTAL		523	48.4%	557	51.6%	1080
						
		Male		Female		TOTAL
BOB Winners		n	%	n	%	
Bloodhound	Giant	3	100.0%	0	0.0%	3
Bullmastiff	Giant	13	92.9%	1	7.1%	14
Central Asian shepherd dog	Giant	0	0.0%	2	100.0%	2
Fox terrier (Smooth)	Toy	15	83.3%	3	16.7%	18
Great Dane	Giant	5	29.4%	12	70.6%	17
Norfolk terrier	Toy	6	60.0%	4	40.0%	10
Norwich terrier	Toy	3	60.0%	2	40.0%	5
Poodle (Toy)	Toy	6	33.3%	12	66.7%	18
Pug	Toy	15	83.3%	3	16.7%	18
Rottweiler	Giant	10	58.8%	7	41.2%	17
Schnauzer (miniature)	Toy	10	66.7%	5	33.3%	15
TOTAL		86	62.8%	51	37.2%	137
						
		Male		Female		TOTAL
BIG Winners or Runners-up		n	%	n	%	
Bloodhound	Giant	0		0		0
Bullmastiff	Giant	0		0		0
Central Asian Shepherd Dog	Giant	0		0		0
Fox terrier (Smooth)	Toy	7	100.0%	0	0.0%	7
Great Dane	Giant	0	0.0%	3	100.0%	3
Norfolk terrier	Toy	0		0		0
Norwich terrier	Toy	0		0		0
Poodle (Toy)	Toy	0		0		0
Pug	Toy	8	100.0%	0	0.0%	8
Rottweiler	Giant	0	0.0%	1	100.0%	1
Schnauzer (miniature)	Toy	0		0		0
TOTAL		15	78.9%	4	21.1%	19
